# Supplementary material for: Life-history stage determines the diet of ectoparasitic mites on their honey bee hosts
Source: Nat Commun. 2024 Jan 25;15:725. doi: 10.1038/s41467-024-44915-x (PMC10811344; doi:10.1038/s41467-024-44915-x)
Supplement: Supplementary file 1 — Supplementary Information [file 41467_2024_44915_MOESM1_ESM.pdf]

Life-history stage determines the diet of ectoparasitic mites on their honey bee hosts

Bin Han, Jiangli Wu, Qiaohong Wei, Fengying Liu, Lihong Cui, Olav Rueppell, Shufa Xu

Supplementary figures and legends

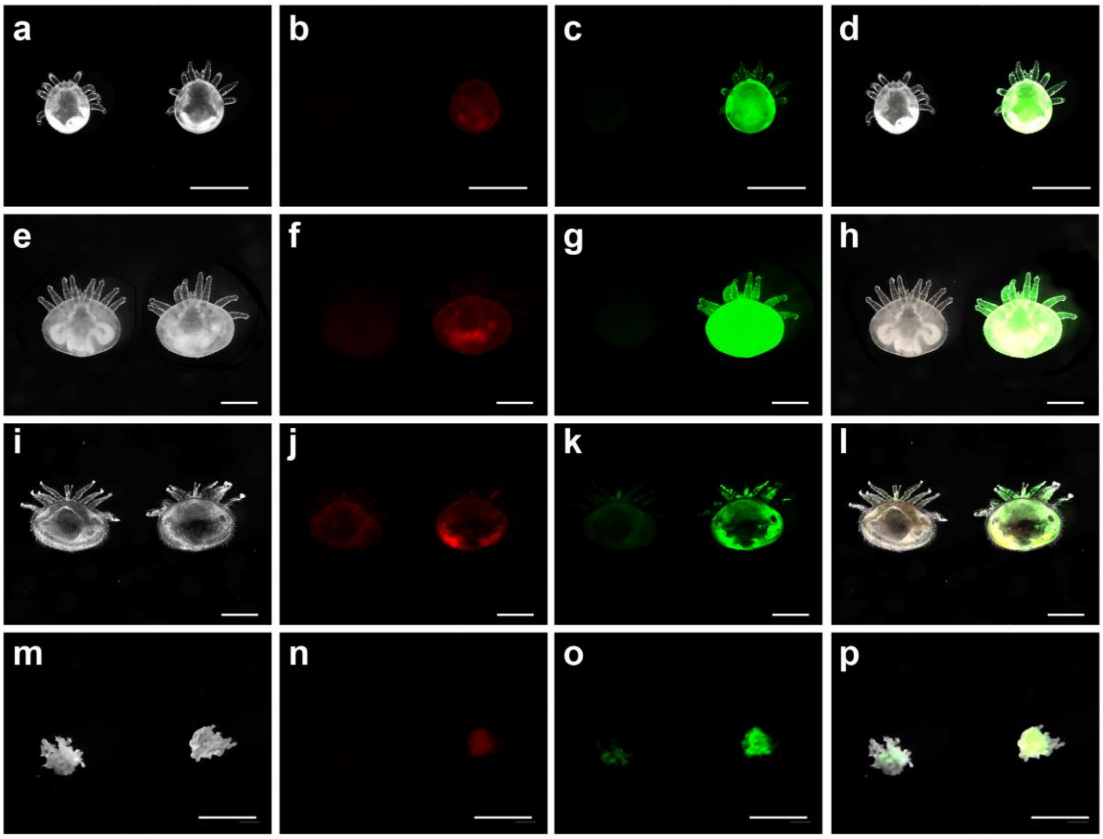

Supplementary Fig. 1 Dorsal views of *Varroa* mites fed on fluorescent biostained honey bee brood. In each photo, the specimen on the left is the control and the one on the right is the treatment. All scale bars represent 1 mm. Dorsal views of protonymphs (**a**–**d**), deutonymphs (**e**–**h**), and foundresses (**i**–**l**) of *Varroa* mites. And *Varroa* feces (**m**–**p**). Each row of photos represents at least 60 samples from six independent experiments.

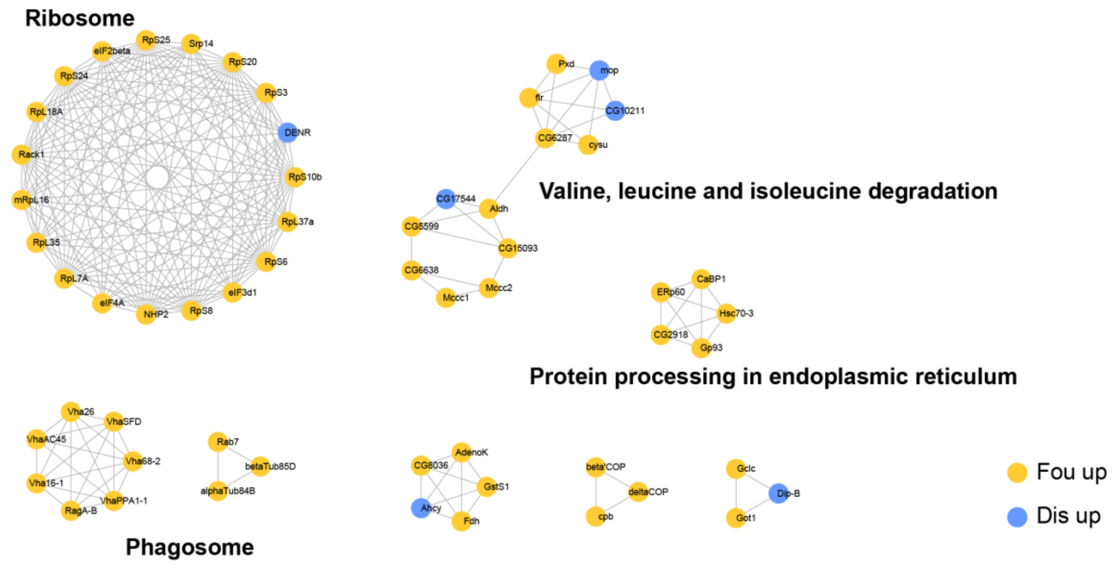

Supplementary Fig. 2 Protein–protein interaction networks. Protein–protein interaction networks were produced based on the differentially expressed *Varroa* proteins between foundresses (Fou) and dispersing mites (Dis). Source data are provided as a Source Data file.

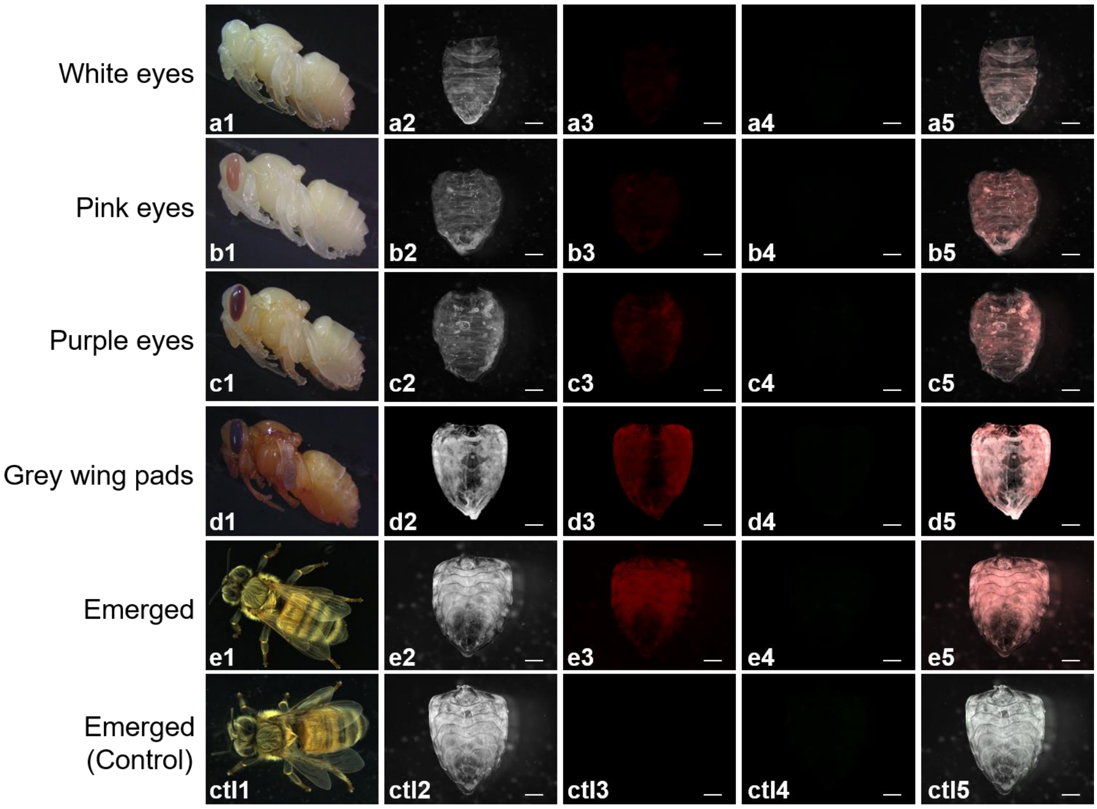

Supplementary Fig. 3 Ventral fat body development in honey bees. Photos show biostained honey bee pupae and adults in brightfield (first), the dissected abdominal sternite in brightfield (second), fluorescence from these abdominal sternite samples associated with Nile red (third) and Uranine (fourth), and all three sternite images merged together (fifth). All scale bars represent 1 mm. Photos of white-eyed pupae (**a1**–**a5**), pink-eyed pupae (**b1**–**b5**), purple-eyed pupae (**c1**–**c5**), grey wing pad pupae (**d1**–**d5**), newly emerged adult bees (**e1**–**e5**), and newly emerged adult control (**ctl1**–**ctl**5). Each row of photos represents at least nine individuals from three independent experiments.

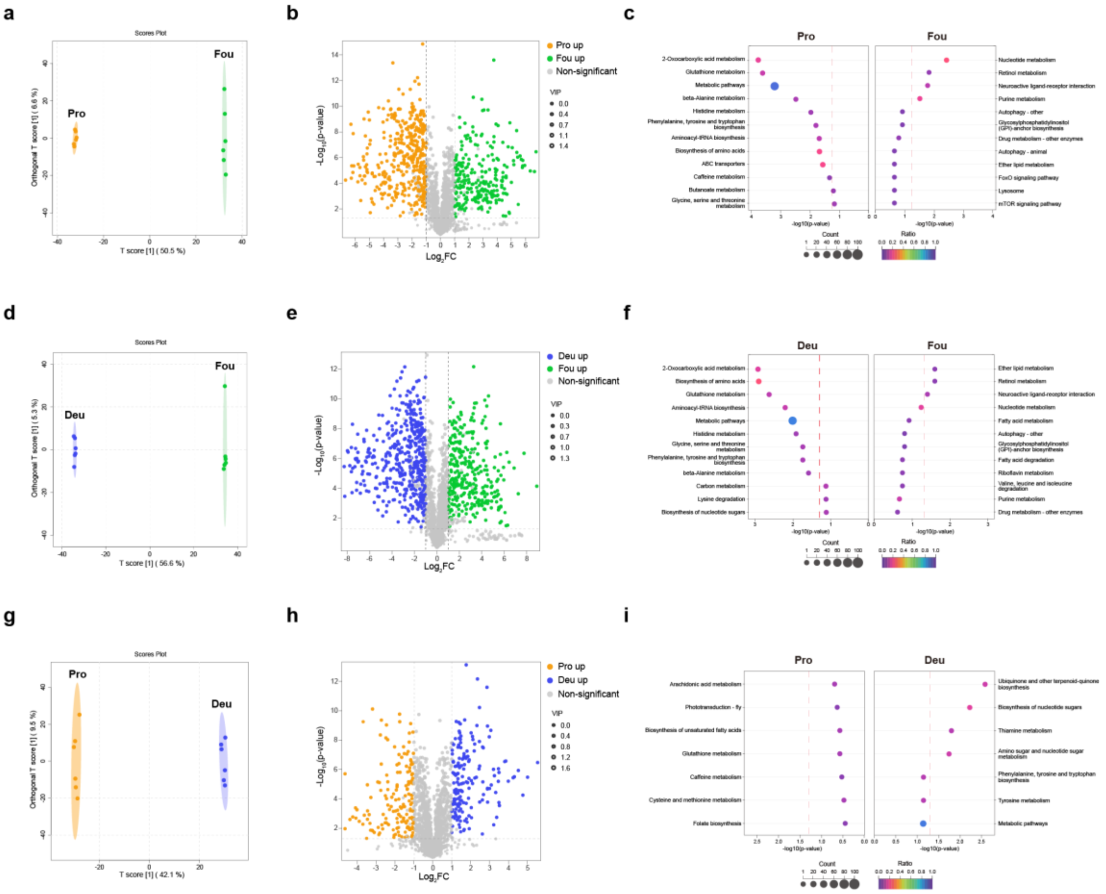

Supplementary Fig. 4 Metabolome comparison among protonymphs (Pro), deutonymphs (Deu), foundresses (Fou) of *Varroa* mites. a–**c** Score plot of orthogonal partial least squares-discriminant analysis (OPLS-DA), volcano plot of metabolite abundance (variable influence on projection (VIP) values from OPLS-DA and p-values from two-sided t-tests), and KEGG pathway enrichment (two-sided hypergeometric tests) in the comparison between Pro and Fou. **d**–**f** Score plot of OPLS-DA, volcano plot of metabolite abundance, and KEGG pathway enrichment in the comparison between Deu and Fou. **g**–**i** Score plot of OPLS-DA, volcano plot of metabolite abundance, and KEGG pathway enrichment in the comparison between Pro and Deu. Difference in metabolite abundance were tested by variable influence on projection (VIP) values derived from OPLS-DA and p-value from two-sided t-tests in (**b**), (**e**), and (**h**). Statistical analyses were performed by two-sided hypergeometric tests in (**c**), (**f**), and (**i**). Source data are provided as a Source Data file.

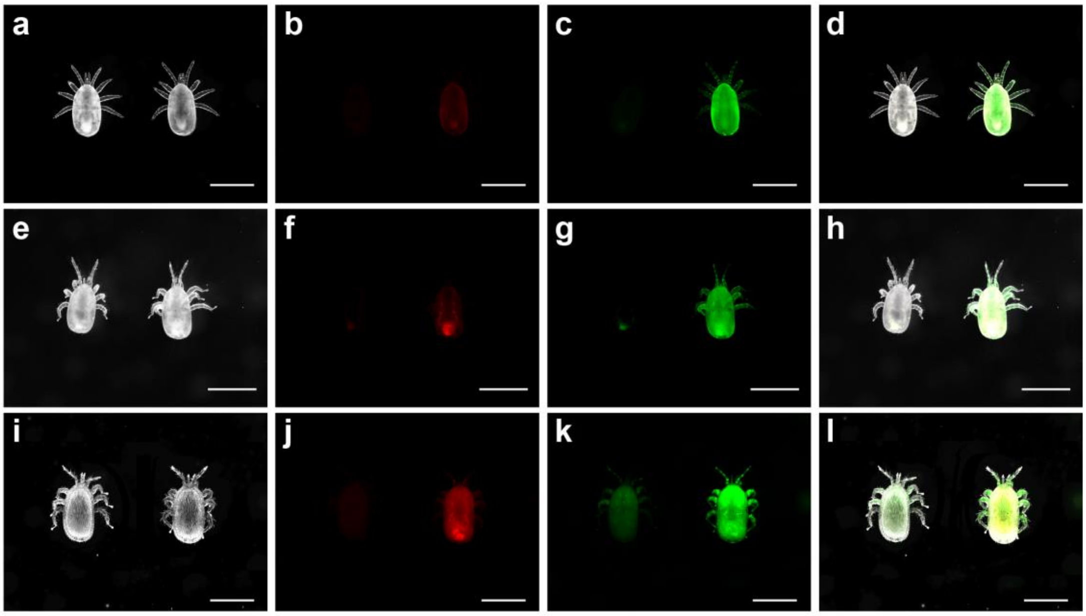

Supplementary Fig. 5 Dorsal views of *Tropilaelaps* mites fed on fluorescent biostained honey bee brood. In each photo, the specimen on the left is the control and the one on the right is the treatment. All scale bars represent 1 mm. Dorsal views of protonymphs (**a**–**d**), deutonymphs (**e**–**h**), and foundresses (**i**–**l**) of *Tropilaelaps* mites. Each row of photos represents at least 60 individuals from six independent experiments.

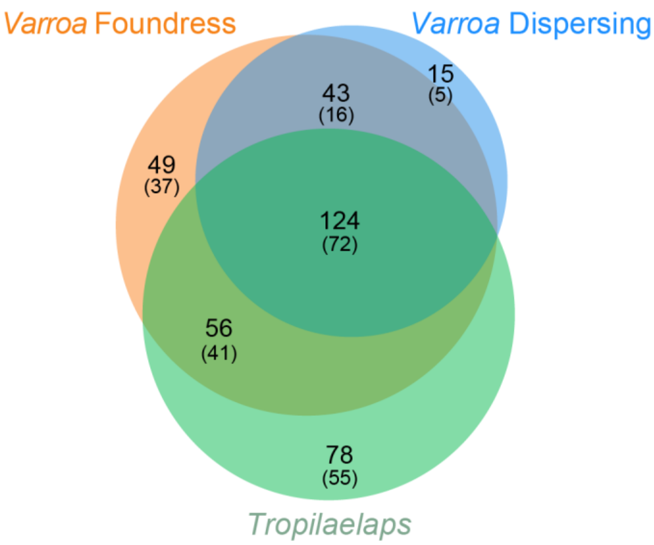

Supplementary Fig. 6 *Tropilaelaps* and *Varroa* proteome overlap analysis. The proteomic profiling of *Tropilaelaps mercedesae* identified 258 honey bee-derived proteins, of which 180 proteins were also found in *Varroa destructor*. In particular, 124 proteins overlapped with proteins found in both foundresses and dispersing *V. destructor*, 56 proteins overlapped with proteins that were only found in *V. destructor* foundresses. The numbers in parentheses represent proteins that had previously been identified in honey bee hemolymph.
