# Supplementary material for: Life-history stage determines the diet of ectoparasitic mites on their honey bee hosts
Source: Nat Commun. 2024 Jan 25;15:725. doi: 10.1038/s41467-024-44915-x (PMC10811344; doi:10.1038/s41467-024-44915-x)
Supplement: Supplementary file 3 — Reporting Summary [file 41467_2024_44915_MOESM3_ESM.pdf]

nature portfolio

Corresponding author(s): Olav Rueppell, Shufa Xu

Last updated by author(s): Nov 27, 2023

Reporting Summary

Nature Portfolio wishes to improve the reproducibility of the work that we publish. This form provides structure for consistency and transparency in reporting. For further information on Nature Portfolio policies, see our Editorial Policies and the Editorial Policy Checklist.

Statistics

For all statistical analyses, confirm that the following items are present in the figure legend, table legend, main text, or Methods section.

n/a	Confirmed	
☐	☒	The exact sample size (*n*) for each experimental group/condition, given as a discrete number and unit of measurement
☐	☒	A statement on whether measurements were taken from distinct samples or whether the same sample was measured repeatedly
☐	☒	The statistical test(s) used AND whether they are one- or two-sided<br>*Only common tests should be described solely by name; describe more complex techniques in the Methods section.*
☐	☒	A description of all covariates tested
☐	☒	A description of any assumptions or corrections, such as tests of normality and adjustment for multiple comparisons
☐	☒	A full description of the statistical parameters including central tendency (e.g. means) or other basic estimates (e.g. regression coefficient) AND variation (e.g. standard deviation) or associated estimates of uncertainty (e.g. confidence intervals)
☐	☒	For null hypothesis testing, the test statistic (e.g. *F*, *t*, *r*) with confidence intervals, effect sizes, degrees of freedom and *P* value noted<br>*Give P values as exact values whenever suitable.*
☒	☐	For Bayesian analysis, information on the choice of priors and Markov chain Monte Carlo settings
☐	☒	For hierarchical and complex designs, identification of the appropriate level for tests and full reporting of outcomes
☒	☐	Estimates of effect sizes (e.g. Cohen's *d*, Pearson's *r*), indicating how they were calculated

Our web collection on statistics for biologists contains articles on many of the points above.

Software and code

Policy information about availability of computer code

Data collection	Fluorescent pictures were captured and processed using LASX software (v2.02.15022). The raw data of proteomics were retrieved using Xcalibur software (v3.0, Thermo Fisher Scientific). The extracted MS/MS spectra were searched against a protein database using MaxQuant software (v.1.6.17.0). The raw data of Metabolomic generated by LC-MS was converted into mzML format by ProteoWizard software (v.3.0.4416).
Data analysis	Protein quantitation analysis was performed using Perseus software (v1.6.2.3). Hierarchical clustering was performed using TBtools software (v1.1043). Functional enrichment analyses, including Gene Ontology (GO) enrichment, Kyoto Encyclopedia of Genes and Genomes (KEGG) pathway enrichment, and protein–protein interaction enrichment were performed using Metascape (v3.5.20230501). Unsupervised PCA (principal component analysis) and supervised orthonormal partial least-squares discriminant analysis (OPLS-DA) were performed with the software package SIMCA (v14.1, Umetrics AB, Sweden). All statistical tests were performed in SPSS (v20.0, IBM).

For manuscripts utilizing custom algorithms or software that are central to the research but not yet described in published literature, software must be made available to editors and reviewers. We strongly encourage code deposition in a community repository (e.g. GitHub). See the Nature Portfolio guidelines for submitting code & software for further information.

Data

Policy information about availability of data

All manuscripts must include a data availability statement. This statement should provide the following information, where applicable:

- Accession codes, unique identifiers, or web links for publicly available datasets
- A description of any restrictions on data availability
- For clinical datasets or third party data, please ensure that the statement adheres to our policy

The proteomic data generated in this study have been deposited in the Proteome Xchange Consortium with the dataset identifier PXD047191 (https://www.iprox.cn//page/SCV017.html?query=IPX0005580000). All data supporting the findings of this study are available in the manuscript or supplementary information. Source data are provided with this paper.

Research involving human participants, their data, or biological material

Policy information about studies with human participants or human data. See also policy information about sex, gender (identity/presentation), and sexual orientation and race, ethnicity and racism.

Reporting on sex and gender	n/a
Reporting on race, ethnicity, or other socially relevant groupings	n/a
Population characteristics	n/a
Recruitment	n/a
Ethics oversight	n/a

Note that full information on the approval of the study protocol must also be provided in the manuscript.

Field-specific reporting

Please select the one below that is the best fit for your research. If you are not sure, read the appropriate sections before making your selection.

[X] Life sciences   [ ] Behavioural & social sciences   [ ] Ecological, evolutionary & environmental sciences

For a reference copy of the document with all sections, see nature.com/documents/nr-reporting-summary-flat.pdf

Life sciences study design

All studies must disclose on these points even when the disclosure is negative.

Sample size	Although we didn't calculate the sample size, we collected as many samples as possible based on the amount of sample needed for each individual experiment. For example, six replicates (20 mg for each replicate) were prepared for the metabolomic analysis, and three additional replicates (50 mg for each replicate) of adult reproducing mites (foundresses) and dispersing mites for proteomic analysis.
Data exclusions	No data were excluded from the analyses.
Replication	Each experiment contained at least three biological replicates, the exact number of which is described in the paper. All attempts at replication were successful.
Randomization	Samples were allocated into experimental groups randomly, and the covariate is not relevant to this study.
Blinding	The investigators were blinded to group allocation during data collection and analysis.

Reporting for specific materials, systems and methods

We require information from authors about some types of materials, experimental systems and methods used in many studies. Here, indicate whether each material, system or method listed is relevant to your study. If you are not sure if a list item applies to your research, read the appropriate section before selecting a response.

Materials & experimental systems

n/a	Involved in the study
☒	☐ Antibodies
☒	☐ Eukaryotic cell lines
☒	☐ Palaeontology and archaeology
☐	☒ Animals and other organisms
☒	☐ Clinical data
☒	☐ Dual use research of concern
☒	☐ Plants

Methods

n/a	Involved in the study
☒	☐ ChIP-seq
☒	☐ Flow cytometry
☒	☐ MRI-based neuroimaging

Animals and other research organisms

Policy information about studies involving animals; ARRIVE guidelines recommended for reporting animal research, and Sex and Gender in Research

Laboratory animals	This study did not involve laboratory animals.
Wild animals	Honeybee worker larvae of one day old were used for in vitro rearing. Foundress mites, both Varroa destructor and Tropilaelaps mercedesae, were collected directly from the sealed honey bee brood cells that had been capped for approximately 24 hours. At the end of the experiment, individuals that were not collected were discarded because they would not survive in the wild.
Reporting on sex	In this study, honeybee worker larvae were used for breeding mites, so they were females. And the bee mites collected were also females.
Field-collected samples	In vitro honey bee larvae rearing was performed using a standard protocol. Briefly, queens were caged on an empty comb to lay eggs. Newly hatched worker larvae were grafted into sterilized 48-well cell culture plates containing plastic queen cups and reared at 34.5°C and 95% RH in a dark incubator.<br>Subsequently, the in vitro reared larvae were used for the mite feeding experiments according to the previously established methods. In short, foundress mites were collected directly from the sealed brood cells that had been capped for approximately 24 hours. The in vitro reared larvae were placed into size “0” gelatin capsules (7.34 mm diameter, Electron Microscopy Sciences, USA) with small custom-made ventilation holes. Foundress mites were individually transferred into a gelatin capsule containing one in vitro reared larva (one mite per capsule), and placed in an incubator at 34.5°C, 75% RH in the dark. The protonymphs, deutonymphs, and adult mites were collected prior to worker emergence, and stored at -80°C for further examination.
Ethics oversight	No ethical approval or guidance was required, as the objects of this study were bees and bee mites, and no transgenic manipulation was carried out.

Note that full information on the approval of the study protocol must also be provided in the manuscript.
