# Supplementary material for: Life-history stage determines the diet of ectoparasitic mites on their honey bee hosts
Source: Nat Commun. 2024 Jan 25;15:725. doi: 10.1038/s41467-024-44915-x (PMC10811344; doi:10.1038/s41467-024-44915-x)
Supplement: Supplementary file 4 — Description of Additional Supplementary Files [file 41467_2024_44915_MOESM4_ESM.docx]

**Description of Additional Supplementary Files**

**Supplementary Data 1:** Proteomics data of *Varroa destructor*.

**Supplementary Data 2:** Proteomics data of *Tropilaelaps mercedesae*.

**Supplementary Data 3:** Metabolomics data of *Varroa destructor* in positive (Sheet1) and negative (Sheet2) modes.
